# Assessment of aquatic food web and trophic niche as a measurement of recovery function in restored mangroves in the Southern Gulf of Mexico

**DOI:** 10.7717/peerj.15422

**Published:** 2023-06-06

**Authors:** Miriam Soria-Barreto, Rosela Pérez-Ceballos, Arturo Zaldívar-Jiménez, Rolando Gelabert Fernández

**Affiliations:** 1 Centro de Investigación de Ciencias Ambientales, Facultad de Ciencias Naturales, Universidad Autónoma del Carmen, Ciudad del Carmen, Campeche, Mexico; 2 Laboratorio de Ecología Acuática y Monitoreo Ambiental, CEDESU, Universidad Autónoma de Campeche, San Francisco de Campeche, Campeche, Mexico; 3 CONACYT Instituto de Ciencias del Mar y Limnología Estación El Carmen UNAM, Universidad Nacional Autónoma de México, Ciudad del Carmen, Campeche, Mexico; 4 ATEC Asesoría Técnica y Estudios Costeros SCP, Mérida, Yucatán, Mexico

**Keywords:** Gulf of Mexico, Fish, Macroinvertebrates, Stable isotopes, Mixing models, Restoration, Seasonal

## Abstract

Mangroves are coastal wetlands with high biodiversity and productivity, with great interaction with coastal environments. In the face of worldwide mangrove loss, restoration projects attempt to recover ecosystem composition and functioning over time. Our objective was to examine and compare the food webs in mangrove areas with different restoration times and in a reference mangrove in Términos Lagoon, Mexico. We estimated the trophic structure, identified the carbon resources that maintain aquatic consumers through the analysis of stable isotopes, and compared the trophic niche of the restored mangroves with the reference mangrove. We analyzed environmental variables, trophic structure, and contributions of resources during three seasons: rainy, dry, and “nortes”. Environmental changes and food structure changed in response to regional seasons. Bayesian mixing models indicated that food webs varied seasonally as a response to the primary productivity developed at Términos Lagoon. As expected, the assimilation of C_3_ plants in the reference mangrove was highest, as a primary (“nortes” season) and secondary resource (dry and rainy seasons). The restored mangroves depended mainly on allochthonous resources (seagrass, epiphytes, and phytoplankton). The assimilation of these resources highlighted the importance of connectivity and the input of sources of carbon from nearby coastal environments. Trophic niche analysis showed that the area with longer restoration time was more similar to the reference mangrove, which is evidence of the importance and efficacy of the restoration process, as well as the restoration of the ecosystem function over time.

## Introduction

Mangrove ecosystems are coastal wetlands distributed in tropical and subtropical intertidal zones (Hopkinson et al., 2018). Recognized for their high biological diversity, they play a fundamental role in providing food, habitat, and protection against predators for a wide variety of terrestrial and aquatic species (Nagelkerken et al., 2008). They are nurseries for various fish and invertebrate species (Beck et al., 2001). In addition, they are vital locations in the life cycle and migratory routes of fish species, many of them commercially valuable, which is why fishing is common in these ecosystems (Blaber, 2007; Blaber, 2013). Mangroves also provide environmental services to human populations, including nutrient regulation, coastal protection, carbon sequestration, and water supply, among others (Lee et al., 2014; Himes-Cornell, Grose & Pendleton, 2018).

Mangroves are highly productive ecosystems, with close and complex interactions with freshwater environments and other coastal ecosystems such as marshes, coral reefs, and seagrasses (Bouillon, Connolly & Lee, 2008; Hopkinson et al., 2018). Mangrove ecosystems are considered to make substantial contributions of organic matter and nutrients to nearby coastal ecosystems, supporting increased productivity and maintaining food webs (Fleming, Lin & Da Silveira Lobo Sternberg, 1990; Lee, 1995; Lee et al., 2014). However, mangrove ecosystems also receive important allochthonous sources of carbon from adjacent marine environments such as seagrasses (Connolly, Gorman & Guest, 2005; Bouillon et al., 2007; Igulu et al., 2013), vegetation in mudflats (Kruitwagen et al., 2010), marine phytoplankton (Bouillon et al., 2007), among others. This exchange of organic matter occurs through lateral and horizontal flows, depending on mangrove geomorphology, communication with the open sea, and tidal conditions (Bouillon et al., 2007; Santos et al., 2021).

Mangrove food webs are complex and dynamic. Their structure varies spatially and temporally depending on environmental characteristics, nutrient availability, changes in the composition and abundance of consumers, among other factors (Abrantes et al., 2015; Claudino et al., 2015a; Faye et al., 2011; Melville & Connolly, 2003). Some studies indicate that mangroves are the main source of carbon that maintains consumer biomass (fish, crabs, mollusks), and this carbon is assimilated and incorporated into food webs as detritus (Abrantes et al., 2015; Abrantes & Sheaves, 2008; Mendoza-Carranza et al., 2010; Muro-Torres et al., 2020). Moreover, mangrove ecosystems develop other basal resources, such as filamentous algae and microphytobenthos, that are important in maintaining food webs and depend on the structural complexity of mangrove trees (Laegdsgaard & Johnson, 2001; Sheridan & Hays, 2003; Giarrizzo, Schwamborn & Saint-Paul, 2011; Whitfield, 2017). Other important allocthonous basal resources include marine phytoplankton, benthic algae, and organic matter (*e.g.*, seagrass) from other coastal ecosystems (Kruitwagen et al., 2010; Vaslet et al., 2012; Claudino et al., 2015b; Sepúlveda-Lozada et al., 2015).

Rapid and accelerated loss of mangroves worldwide (Romañach et al., 2018) has driven projects aimed at their restoration and rehabilitation (López-Portillo et al., 2017). Studies have shown changes in soil physicochemical parameters, mangrove structure, composition, diversity, and structure of consumer communities throughout the restoration process (Bosire et al., 2008; Zhao et al., 2016; Salmo III, Tibbetts & Duke, 2018). Restored mangrove function is manifested in nutrient processing, biological interactions, and trophic dynamics, which are indicators of the reactivation of processes and functions (Bosire et al., 2008).

Stable isotopes (*δ*^13^C and *δ*^15^N) can be used to evaluate the functioning of aquatic ecosystems by analyzing of flow of nutrients or energy over food webs (Layman et al., 2012). They can also be used to determine the source of nutrients and organic matter assimilated by consumers in the food webs, whether it comes from mangrove organic matter or another basal resource in the ecosystem (Layman, 2007; Dittmar, Koch & Jaffé, 2009); and to compare food web structure and resource use along an environmental gradient (Layman et al., 2007). Isotopes are a useful tool in ecological restoration, as they can help us understand the functioning of ecosystems and their response to changes in the environment. Studies of trophic ecology in restored mangroves have focused on macroinvertebrates, showing differences in assimilation depending on the age of the mangroves and their position in the intertidal zone (Van Hieu et al., 2020; Then et al., 2021). Recently, through the calculation of hypervolumes, the response of the energy flow in restored mangroves has been estimated, showing the levels of recovery of the food web function and the success of the restoration (James et al., 2020).

Términos Lagoon is a lagoon system in the southern Gulf of Mexico known for its high biodiversity and variety of habitats, including extensive mangrove areas, seagrass beds, areas of freshwater influence, and others with marine influence (Herrera-Silveira et al., 2019). Its trophic dynamics vary seasonally and depend on mangrove detritus and development of phytoplankton and seagrass (Yáñez Arancibia et al., 2013; Sepúlveda-Lozada et al., 2015). Mangrove restoration projects have been implemented in Términos Lagoon, based on hydrological and sedimentological restoration, through the opening of tidal channels with subsequent reforestation with selected species and the participation of the local population (Agraz-Hernández et al., 2010; Zaldívar-Jiménez et al., 2017).

Evaluations of the restoration process in the mangroves of Términos Lagoon have documented the biogeochemical response of the soil with decreasing salinity and sulfide concentrations, and the establishment and growth of mangrove seedlings (Pérez-Ceballos et al., 2018; Pérez-Ceballos et al., 2020). Changes in the composition of mangrove-associated ichthyofauna related to mangrove conservation state (Soria-Barreto et al., 2021), changes in fish parasite communities (Morales-Serna et al., 2019), and avifauna (Canales-Delgadillo et al., 2019) have also been reported. Additional research is needed describing and monitoring how restoration processes affect habitat changes and the aquatic community, including functional and trophic descriptors.

The present study objective was to evaluate and compare the food web structure in mangrove communities with different restoration times and seasonality. We aimed to determine the basal resources which maintain fish and macroinvertebrate communities in these areas and compare the trophic niche between the reference mangrove and the restored ones. Changes in trophic structure were expected to be related to changes in environmental conditions and consumer composition during the restoration process (Bosire et al., 2008; Salmo III, Tibbetts & Duke, 2018). We expected that mangroves would be the main carbon source in the reference mangrove (Abrantes et al., 2015; Abrantes & Sheaves, 2008; Mendoza-Carranza et al., 2010; Muro-Torres et al., 2020), and that other basal resources would contribute to food webs seasonally (Yáñez Arancibia et al., 2013; Herrera-Silveira et al., 2019); while in restoration areas the resources would be modulated by the time of restoration. We expected that consumer trophic niche from the area with the longest restoration period would be similar to that in the reference mangrove, as a result of the mangrove structure and the reestablishment of ecosystem function (James et al., 2020; Then et al., 2021).

## Materials & Methods

### Ethical statement

The care and use of animals complied with the Comisión Nacional de Acuacultura y Pesca (CONAPESCA) guidelines and policies. Sampling was carried out with the permit number DF00000156-C from CONAPESCA.

### Study area

**Figure 1:**
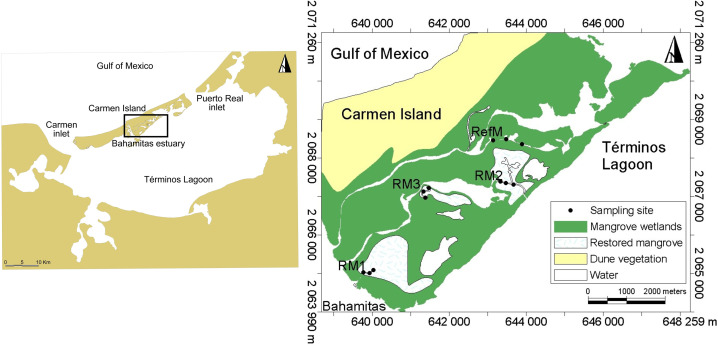
Location of sampling sites in Términos Lagoon. RM1, mangrove restored in 2010; RM2, mangrove restored in 2014; RM3, mangrove restored in 2018; RefM, reference mangrove.

Located in the southern Gulf of Mexico in the state of Campeche, Mexico, Términos Lagoon is formed by a barrier island and a coastal lagoon system (Fig. 1). It encompasses 120,000 hectares of mangrove, as well as priority sites of biological relevance requiring ecological restoration (Rodríguez-Zúñiga et al., 2013). Characterized by high productivity, biological diversity, and important fisheries, it is a Federal natural protected area for flora and fauna (Yáñez Arancibia et al., 2013). Seawater constantly flows into the lagoon due to mixed diurnal tides with an average amplitude of 0.43 m (range 0.3 to 0.7 m) (Yáñez Arancibia & Day, 2005). There are three climatic seasons in the area. The “nortes” season from October to February is characterized by intermittent rains from winter storms and winds >8 m s^−1^. The dry season from March to May is characterized by minimal or no precipitation. The rainy season occurs from June to September, with rainfall of 180 mm per month (Yáñez Arancibia & Day, 1982; Yáñez Arancibia & Day, 2005).

Sampling was done on the inland coast of Carmen Island in the Bahamitas Estuary, in four areas with tidal mangrove channels: one reference and three restored (Fig. 1). The latter three channels were dredged by tracing the base preferential flows, which were generated by microwatershed modeling analysis with microtopography information (Pérez-Ceballos et al., 2020). Restoration allowed the recovery of hydrological connectivity with Términos Lagoon, improved the biogeochemical quality, and favored the natural regeneration of the mangroves (Zaldívar-Jiménez et al., 2017).

We selected one area with natural mangroves without apparent modifications, known as reference mangrove (RefM) (18.7028 N, −91.6424 W), where vegetation was dominated by red mangrove (*Rhizophora mangle*) along the channel edges, with white mangrove (*Laguncularia racemosa*) and black mangrove (*Avicennia germinans*) trees and shrubs in the interior. We selected three areas with different restoration times; the area with the longest time since restoration is called RM1 (18.6721 N, −91.6721 W), which was restored in 2010–2011. It is 320 m long and harbors red, white, and black mangrove, although red mangrove dominates. The second area restored is RM2 (18.6928 N, −91.6411 W), which was restored in 2014. It is 1,000 m long, and harbors all three mangrove species, although black mangrove shrubs and red mangrove juveniles dominate the area. The third area restored is RM3 (18.6896 N, −91.6596 W), which was restored in 2018. It is 400 m long and contains all three mangrove species, with red mangrove seedlings and juveniles dominating along with some black mangrove shrubs. In the inner littoral of Carmen Island, adjacent to the study area there are seagrass beds, which constitute an important habitat for species in Términos Lagoon (Yáñez Arancibia, Lara-Domínguez & Day Jr, 1993).

### Sampling and laboratory processing

Sampling was done during all three regional climatic seasons: rainy (September 2020); “nortes” (January 2021); and dry (April 2021). Three sampling sites were established in each area to measure environmental parameters. Depth was measured with a graduated ruler. Temperature (°C), dissolved oxygen (DO; mg/L), salinity (ups), total dissolved solids concentration (TDS; mg/L), conductivity (mS/cm), and pH were measured with a YSI Pro multiparameter device. Three water samples were collected in each area to quantify chlorophyll *a* concentration, which was analyzed with the ethanol extraction method and measured with a spectrophotometer (Nusch, 1980).

Samples of available basal resources were collected. Observed C_3_ plants in the restored areas included mangrove (*R. mangle, L. racemosa, A. germinans*) and saltwort (*Batis maritima*). Three sets of leaf samples of each plant species were collected and preserved in salt (Arrington & Winemiller, 2002). Seston was separated by filtering 150 ml water with a previously burned Whatman GF/F filter (0.7 µm). Phytoplankton was collected using a cascade filter consisting of 50, 30, and 15 µm sieves. Large volumes of water were run through the filters until the 15 µm sieve was saturated (Sepúlveda-Lozada et al., 2015). The retained fraction was washed with distilled water and filtered through a previously burned Whatman GF/F filter (0.7 µm).

Fish were caught using different fishing gear. Ten baited minnow traps with fish pellets (57.9 cm in diameter × 22.1 cm in height, 0.5 cm mesh) were placed at the channel banks. Five baited cylindrical traps (60 cm long × 26 cm diameter; one cm mesh) were placed in the middle of the channel. A multi-mesh gillnet (55 m long × 2.5 high; 1-to−3.5-inch mesh) was installed at the channel entrance and a cast net (2 m diam., one cm mesh) used when conditions allowed. Fauna sampling was done in the morning at low tide, for approximately 3 to 4 h. Macroinvertebrates (mollusks and crustaceans) were collected with nets or manually.

Collected specimens were preserved on ice for transport to the laboratory. Muscle tissue samples were extracted from the faunal samples of sufficient size: from the muscular foot in the case of mollusks; from the claws in crabs; and from dorsal muscle tissue in fish. For small fish such as poecilids, a sample composed of 5 to 8 organisms was used (Garcia et al., 2007). Tissue samples were preserved in salt and frozen (Arrington & Winemiller, 2002). Specialized keys were used for taxonomic identification of macroinvertebrates (mollusks and crustaceans) (Leal, 2002; Tavares, 2002), and fish (Castro-Aguirre, Espinosa-Pérez & Schmitter-Soto, 1999; Carpenter Kent, 2002; Miller, 2009).

In the laboratory, the plant and muscle tissue samples were washed with distilled water to remove excess salt. They were oven-dried at 60 °C for 48 h and macerated with a mortar and pestle. Subsamples were weighed out (1.5 to 2.5 mg) and placed in tin capsules. These subsamples were sent to the Isotope Laboratory at the University of California, Davis for analysis of stable carbon (^13^C/^12^C) and nitrogen (^15^N/^14^N) isotopes. Isotopic values were expressed in parts per thousand (‰) relative to standard values in delta (*δ*) notation. The value is calculated using the following formula:

\begin{eqnarray*}\delta X=[(\text{R sample}/\text{R standard})]-1\times 1{0}^{3} \end{eqnarray*}

where X is ^15^N or ^13^C, and R is the ^13^C/^12^C or ^15^N/^14^N ratio, using Pee Dee Belemnite as the standard value for carbon, and atmospheric nitrogen for nitrogen. Analytical precision was ± 0.04 for both measurements *δ*^13^C and *δ*^15^N. Mean SD for the reference materials was ± 0.07‰ for *δ*^13^C values, and ± 0.05‰ for *δ*^15^N values. The reference materials used for each isotope are listed in (Supplementary Material S1, T1).

### Data analysis

Environmental patterns in the tidal channels were elucidated with a principal components analysis (PCA) incorporating the environmental variables documented in the field, with the values log-transformed (x+1) (Supplementary Material S1, T2). The analysis was done with the R ver. 3.6.3 software program (R Core Team, 2021), using the correlation matrix in the vegan package (Oksanen et al., 2019). Interpretation of the PCA was based on those variables with loadings greater than 0.6 (Sedeño Díaz & López-López, 2007).

Identification of the differences in environmental variables between seasons and areas was done with a non-parametric Kruskal-Wallis analysis. This was done because the data did not meet normality and homoscedasticity assumptions. When significant differences were detected, a multiple comparison was made with Dunn’s test with Bonferroni’s correction, with the dunn.test package (Dinno, 2015).

For the basal resources, the *δ*^13^C and *δ*^15^N values (Supplementary Material S1, T3) were compared between seasons and areas with a non-parametric Kruskal-Wallis analysis and a multiple comparison including Dunn’s test with Bonferroni’s correction. The *δ*^13^C values of consumers tissues (Supplementary Material S1, T4) were corrected for lipid content according to the mathematical equation provided by Post et al. (2007). Trophic structure at each area was graphed as a biplot using the *δ*^13^C and *δ*^15^N values for fish, macroinvertebrates and basal resources, including periphyton and seagrass as reported for Términos Lagoon (Sepúlveda-Lozada et al., 2015). The *x*-axis represents consumer assimilation by organic carbon source (*δ*^13^C), and the *y*-axis represents trophic level (*δ*^15^N) (Peterson & Fry, 1987).

Consumer trophic structure was estimated and compared through assembly metrics (Layman et al., 2007). The *δ*^15^N range (NR) provides data on a metric that represents the vertical trophic structure of the community. The *δ*^13^C range (CR) provides an estimate of the basal resource diversity utilized by the community. Total convex hull area (TA) measures the total amount of isotopic niche space in the community. Mean distance to the centroid (CD) represents the average degree of diversity of isotopic niches and provides data on species spacing. Mean nearest neighbor distance (MNND) measures relative density and species clustering in isotopic space. The standard deviation of nearest neighbor distance (SDNND) measures spatial density uniformity and packing. The isotopic spaces were estimated for each area and season using Bayesian standard ellipse areas (SEA_B_). The correction for small sample sizes was applied to estimates of standard ellipse areas (SEA_C_). This is a robust method for making statistical comparisons between different groups/samples; in the present case, it allowed comparison of community isotopic niche at different sites and in different seasons. The above metrics were calculated with the SIBER package in R (Jackson et al., 2011).

Basal resource assimilation was estimated with MixSIAR Bayesian mixing models (Stock & Semmens, 2016). The *δ*^13^C and *δ*^15^N values were corrected by considering the trophic level of the species as in the methods of Abrantes et al. (2013). The trophic level considered was 3.0 for piscivores, 2.7 for zoobenthivores, 2.6 for planktivores and omnivores, and 2.4 for detritivores following Sepúlveda-Lozada et al. (2015). The trophic fraction considered per trophic level was 0.4‰ *δ*^13^C and 3.4‰ *δ*^15^N following Post (2002). Prior to analysis of the mixing models, Pearson correlations was performed between *δ*^13^C and *δ*^15^N values of seston and phytoplankton. Because the seston had a significant high correlation with phytoplankton *δ*^13^C (Pearson’s *r* = 0.72, *p* < 0.05) and *δ*^15^N (Pearson’s *r* = 0.65, *p* < 0.05), it was not considered in analysis. Also, we verified that consumers were within the mixing polygon defined by basal resources and considering the selected trophic discrimination factors (Supplementary material S2, Fig. 1) (Smith et al., 2013; Phillips et al., 2014). Estimations were made of the contributions of each basal resource to the biomass of all consumers (fish, crustaceans, and mollusks) in each area during each season. Four basal resources were used in the models: C_3_ plants, phytoplankton and periphyton and seagrass, reported for Términos Lagoon (Sepúlveda-Lozada et al., 2015) (Supplementary Material S1, T5). The models consider estimated trophic fractionation for carbon (0 ± 1.3‰; (Post, 2002) and nitrogen (0 ± 1‰; Vanderklift & Ponsard, 2003). They were run with a chain length of 300,000 iterations, a burn phase of 200,000 iterations and a thinning of 100. The models produced confidence intervals of each basal resource’s contribution, at a 95% credibility value. They exhibited convergence based on the Gelman–Rubin and Geweke diagnosis (Stock & Semmens, 2016).

The results of the mixing analysis were used to estimate the consumer’s niche n-dimensional hypervolume, to compare the niche between reference and restored mangroves. Each niche dimension represented a basal resource used by consumers (Lesser et al., 2020; Rezek et al., 2020). The mixing models provided an estimate of the basal resources used by each consumer, these data were z-transformed prior to hypervolume construction (Blonder et al., 2014). The hypervolume was estimated using Gaussian kernel density with the hypervolume package in R (Blonder et al., 2018). Overlap hypervolume (Sorensen’s index and confidence intervals) was obtained to estimate similarity between reference mangrove with each restored mangrove areas.

## Results

### Environmental analysis

**Figure 2:**
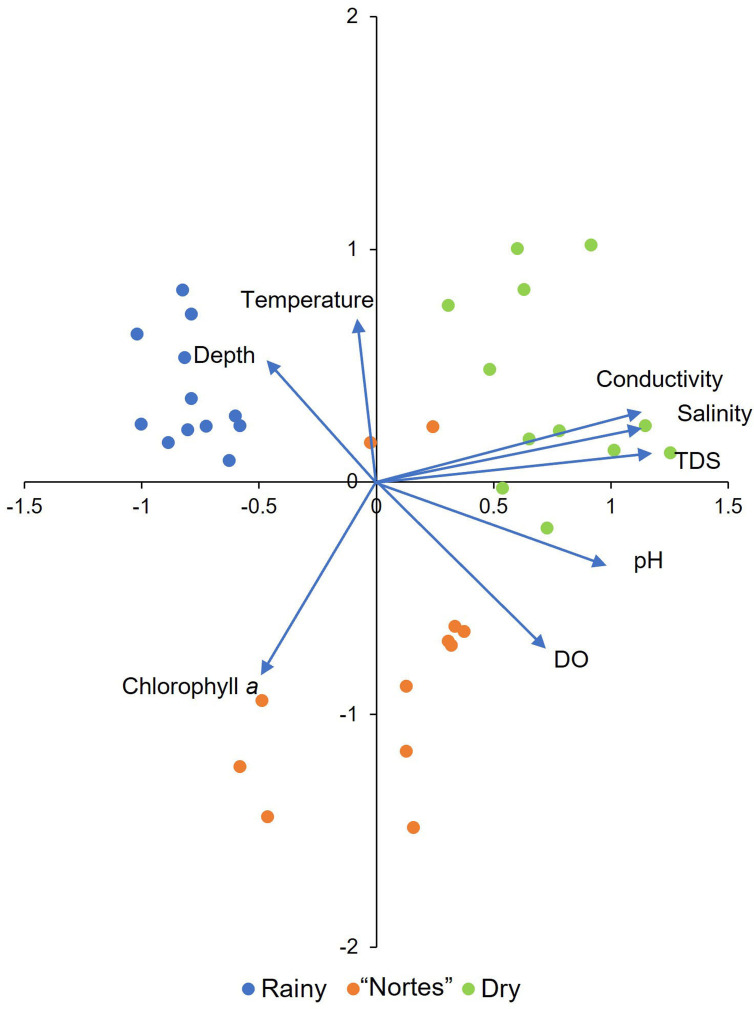
Principal component analysis derived biplot of the environmental parameters of mangroves studied. TDS, total dissolved solids; DO, dissolved oxygen.

The PCA showed that the four analyzed mangroves areas exhibited similar environmental characteristics in response to season. The first two axes explained 68.56% of the variation and all the environmental variables were important (Fig. 2, Supplementary Material S1, T6). We detected environmental differences between seasons. Channel depth was greater in the rainy season (*χ*^2^ = 24.43, *df* = 2, *p* < 0.001), with average values greater than 100 cm (Table 1). Dissolved oxygen (DO) concentrations were low in rainy season (average = 1.1 to 3.1 mg/L, Table 1), with significant differences compared to the “nortes” season (*p* < 0.001). Values for pH were also low in the rainy season, with values from 7.3 to 7.6 (Table 1, *χ*^2^ = 16.81, *df* = 2, *p* < 0.001). Water temperatures were lowest in the “nortes” season, with values from 22.6 to 30.4 °C (Table 1, *χ*^2^ = 13.46, *df* = 2, *p* = 0.001). Conductivity increased during the dry season, with values greater than 60 mS/cm (Table 1, *χ*^2^ = 30.30, *df* = 2, *p* < 0.001). Salinity values ranged from 39.8 to 48.1 ups (Table 1, *χ*^2^ = 30.72, *df* = 2, *p* < 0.001) and TDS concentrations were higher than 38.8 g/L (Table 1, *χ*^2^ = 30.30, *df* = 2, *p* < 0.001).

Differences between mangroves areas were found for DO (*χ*^2^ = 11.69, *df* = 3, *p* = 0.009) and pH (*χ*^2^ = 10.08, *df* = 3, *p* = 0.02). Dissolved oxygen concentration was higher in RM2 than in RM3 (*p* = 0.02) and RefM (*p* = 0.007). Values for pH were also higher in RM2 than in RefM (*p* = 0.02).

### Basal resources

In C_3_ plants, *δ*^13^C average values ranged from −29.3 to −26.4‰ (Supplementary Material S1, T5), with significant differences between seasons (*χ*^2^ = 14.19, *df* = 2, *p* < 0.001) and areas (*χ*^2^ = 21.15, *df* = 3, *p* < 0.001). During the rainy season, C_3_ plants had enriched *δ*^13^C values (*p* < 0.01). The C_3_ plants in RM2 and RM3 were more *δ*^13^C enriched than in RM1 and RefM (*p* < 0.01). Averages values for *δ*^13^C in seston varied between −26.2 and −16.7‰ (Supplementary Material S1, T5), with differences between seasons (*χ*^2^ = 23.19, *df* = 2, *p* < 0.001), ^13^C depleted values in rainy season (*p* < 0.001), and no significant differences between areas. In phytoplankton, *δ*^13^C average values ranged from −24.4 to −18.1‰ (Supplementary Material S1, T5), with significant differences between seasons (*χ*^2^ = 22.82, *df* = 2, *p* < 0.001), ^13^C depleted values in the rainy season (*p* < 0.01), and no differences between areas.

**Table 1:** Mean values (standard deviation) of environmental variables by area and season.

**Season**	**Area**	**Depth (cm)**	**Temperature (°C)**	**TDS (g/l)**	**Conductivity (mS/cm)**	**Salinity (ups)**	**DO (mg/l)**	**pH**	**Chlorophyll** ***a*** **(µg/l)**
Rainy	RM1	110.7 (15.6)	29.6 (0.2)	19.3 (0.2)	38.7 (0.3)	24.1 (0.3)	2.3 (0.9)	7.3 (0.2)	7.2 (0.8)
	RM2	132.7 (4.0)	33.6 (1.2)	18.1 (0.05)	36.1 (0.03)	22.6 (0.01)	3.1 (0.9)	7.6 (0.1)	9.5 (2.5)
	RM3	106.7 (10.4)	31.5 (0.6)	18.9 (0.1)	37.7 (0.2)	23.7 (0.2)	2.2 (0.9)	7.4 (0.1)	7.6 (1.8)
	RefM	118.7 (10.3)	29.0 (0.1)	18.8 (0.2)	37.7 (0.4)	23.8 (0.3)	1.1 (0.2)	7.3 (0.3)	8.6 (3.5)
“Nortes”	RM1	67 (32)	22.6 (0.3)	29.5 (0.1)	43.3 (0.1)	29.4 (0.2)	4.7 (0.3)	7.9 (0.04)	8.9 (1.5)
	RM2	77 (7.9)	25.3 (0.8)	33 (3.2)	48.4 (0.4)	31.4 (0.4)	6.0 (0.6)	7.9 (0.1)	6.9 (0.5)
	RM3	22.7 (4.9)	30.4 (2.8)	30.4 (0.4)	51.6 (3.0)	29.9 (0.9)	4.0 (1.6)	7.5 (0.2)	5.2 (2.4)
	RefM	44.3 (6.8)	27.5 (0.5)	20.2 (1.6)	40.4 (3.4)	25.3 (1.6)	3.5 (0.4)	7.5 (0.2)	20.7 (8.0)
Dry	RM1	77.3 (28)	28.3 (0.4)	40 (0.2)	65.5 (0.9)	41.3 (0.3)	3.7 (0.2)	7.9 (0.04)	7.9 (1.4)
	RM2	81 (9.5)	33.5 (1.6)	45.9 (0.7)	81.7 (1.5)	48.1 (0.8)	7 (2.4)	8.2 (0.1)	7.6 (0.9)
	RM3	59.3 (9.5)	28.7 (1.2)	45.8 (0.6)	75.3 (2.5)	48.1 (0.8)	2.2 (0.8)	7.7 (0.1)	5.1 (2.1)
	RefM	80.7 (7.1)	29.4 (0.6)	38.8 (0.8)	64.6 (1.8)	39.8 (0.9)	2.8 (0.7)	7.7 (0.1)	4.9 (1.5)

**Notes.**

RM1 mangrove restored in 2010 RM2 mangrove restored in 2014 RM3 mangrove restored in 2018 RefM reference mangrove TDS total dissolved solids DO dissolved oxygen

Average values for *δ*^15^N in C_3_ plants varied between −2.5 and 3.8‰ (Supplementary Material S1, T5), with significant differences between areas (*χ*^2^ = 12.59, *df* = 3, *p* < 0.001). Differences were observed between RefM and RM1 (*p* = 0.02), and between RefM and RM3 (*p* = 0.002). No differences in *δ*^15^N values were observed between seasons. Seston *δ*^15^N average values varied from 1.0 to 3.5‰ (Supplementary Material S1, T5), with significant differences between seasons (*χ*^2^ = 20.24, *df* = 2, *p* < 0.001), and enriched values during the rainy season (*p* < 0.001). Values for *δ*^15^N in phytoplankton ranged from 2.1 to 4.4‰ (Supplementary Material S1, T5), with differences between seasons (*χ*^2^ = 12.91, *df* = 2, *p* = 0.002), and enriched values during the rainy season (*p* < 0.01). No differences in *δ*^15^N values were observed between areas in seston and phytoplankton.

### Consumers

**Figure 3:**
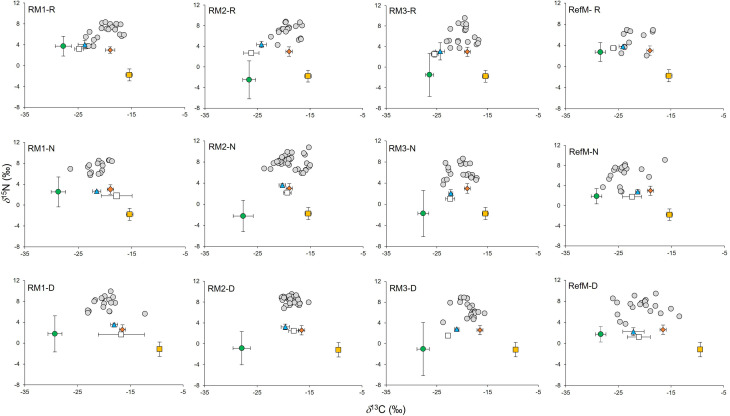
Biplot of *δ*^13^C and *δ*^15^N values of consumers and their basal resources (mean ±SD) in mangrove areas. Gray circles represent consumers. Green circles represent *C*_3_ plants. White squares represent seston. Blue triangles represent phytoplankton. Orange diamonds represent epiphytes. Yellow squares represent seagrass. RM1, mangrove restored in 2010; RM2, mangrove restored in 2014; RM3, mangrove restored in 2018; RefM, reference mangrove. R, rainy season; N, “nortes” season; and D, dry season.

The consumers samples were composed mainly of fish represented by 22 species, (mostly resident species), mollusks (three species) and crustaceans (five species) (Supplementary Material S1, T4). In consumers, *δ*^13^C values ranged from −25.3 to −16.6‰ in the rainy season, from −28.2 to −15.0‰ in the “nortes” season and from −26.5 and −12.2‰ in the dry season (Fig. 3). Differences were observed between seasons (*χ*^2^ = 14.18, *df* = 2, *p* < 0.001), with enriched values in the dry season (*p* < 0.01). Differences were also observed between areas (*χ*^2^ = 52.66, *df* = 3, *p* < 0.001), with ^13^C depleted values in the RefM (*p* < 0.001).

Values for *δ*^15^N in consumers ranged from 2.0 to 9.6‰ in the rainy season, from 2.8 to 10.8‰ in the “nortes” season, and from 3.7 to 9.9‰ in the dry season (Fig. 3). Differences were observed between seasons (*χ*^2^ = 19.01, *df* = 2, *p* < 0.001), with *δ*^15^N depleted values in the rainy season (*p* = 0.01). Differences were also observed between areas (*χ*^2^ = 48.62, *df* = 3, *p* < 0.001), with enriched values in RM3 (*p* = 0.001).

### Trophic structure

**Figure 4:**
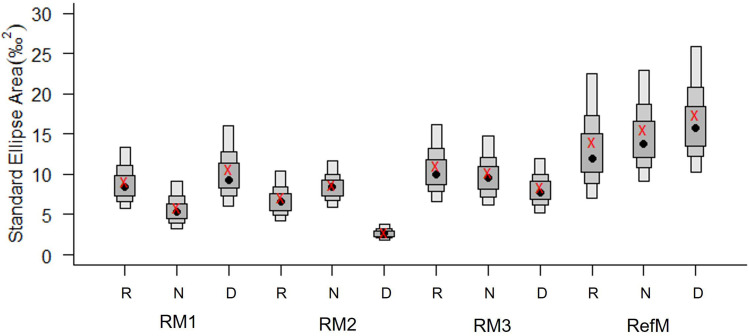
Bayesian standard ellipse areas (SEA, ‰^2^) of the consumers in restored and reference mangroves. The boxes represent 50, 75, and 95% of the Bayesian credible intervals of SEA_*B*_. The black points represent the average area of SEA_*B*_ and the red crosses represent the average, calculated using the sample size correction (SEA_*C*_). RM1, mangrove restored in 2010; RM2, mangrove restored in 2014; RM3, mangrove restored in 2018; RefM, reference mangrove. R, rainy season; N, “nortes” season; and D, dry season.

Consumers in the RefM (fish, mollusks, and crustaceans) used the widest diversity of *δ*^13^C and *δ*^15^N basal resources (CR, NR, Table 2). This community also exhibited the highest trophic diversity (TA, CD, Table 2, SEA_C_ Fig. 3) and greatest trophic niche dispersion (MNND, SDNND, Table 2). Among the restored areas, the community of RM1 exhibited the highest values of CR, TA, CD and MNND *versus* the other restored areas (Fig. 4, Table 2).

**Table 2:** Layman community metrics obtained for each restored mangrove areas (RM1, RM2, and RM3), and the reference mangrove (RefM).

**Metric**	**RM1**	**RM2**	**RM3**	**RefM**
NR	1.08	1.27	0.33	1.64
CR	1.75	0.65	1.54	3.34
TA	0.94	0.06	0.05	2.40
CD	0.91	0.53	0.61	1.50
MNND	1.31	0.64	0.68	2.15
SDNND	0.39	0.26	0.40	0.23

**Notes.**

NR *δ*^15^N range CR *δ*^13^C range TA total area of the convex hull CD mean distance to the centroid MNND mean nearest neighbor distance SDNND standard deviation of the nearest neighbor distance

Trophic diversity in terms of SEA_C_ varied between seasons and areas (Fig. 4). The RefM had the highest values in all three seasons, with increases during the dry season. The restored areas had a different pattern, with notably low trophic diversity in RM2, especially during the dry season.

### Basal resource contributions

Based on the MixSIAR mixing models, the basal resources contributing to consumer biomass varied between areas and seasons (Table 3). During the rainy season, seagrass and epiphytes constituted the most important resources. The mollusks of all restored areas assimilated seagrass as a main resource, on average from 59% to 77%; C_3_ plants were the second most assimilated carbon resource in RM1 and RM3 (19% and 15%, respectively), and epiphytes in RM2 (9%). Crustaceans and fish of all restored areas assimilated mainly epiphytes (from 37% to 72%); phytoplankton was the second most assimilated carbon resource (from 12% to 31%); except for fish of RM1 where seagrass was the second carbon resource (26%). In the reference mangrove, consumers assimilated mainly seagrass (from 40% to 72%) and C_3_ plants were the second most assimilated carbon resource (from 15% to 39%).

**Table 3:** Estimated percent contributions of basal resources to consumers per area and season. Values indicate median contribution with the 95% Bayesian credibility interval. Major contributions are shown in bold.

Rainy season	Consumer	C_3_ plants	Phytoplankton	Seagrass	Epiphytes
RM1	Mollusk	19 (0-42)	11 (0-42)	**62 (40-81)**	8 (0-34)
	Crustacean	14 (0-61)	19 (0-80)	14 (0-42)	**53 (0-100)**
	Fish	10 (0-29)	22 (0-57)	26 (0-44)	**42 (1-94)**
RM2	Mollusk	8 (0-26)	6 (0-23)	**77 (52-100)**	9 (0-36)
	Crustacean	6 (0-22)	26 (0-60)	22 (0-48)	**46 (0-100)**
	Fish	6 (0-22)	12 (0-57)	10 (0-43)	**72 (2-100)**
RM3	Mollusk	16 (0-40)	14 (0-40)	**59 (31-86)**	11 (0-39)
	Crustacean	12 (0-40)	31 (0-81)	20 (0-48)	**37 (0-98)**
	Fish	10 (0-27)	30 (1-59)	18 (0-40)	**42 (3-80)**
RefM	Mollusk	15 (0-46)	7 (0-26)	**72 (33-100)**	6 (0-28)
	Crustacean	39 (4-69)	8 (0-32)	**48 (21-71)**	5 (0-24)
	Fish	28 (0-54)	21 (0-53)	**40 (24-53)**	11 (0-41)
“Nortes” season	Consumer	C_3_ plants	Phytoplankton	Seagrass	Epiphytes
RM1	Crustacean	17 (0-46)	**57 (1-100)**	8 (0-25)	18 (0-67)
	Fish	15 (0-39)	**59 (4-100)**	9 (0-24)	17 (0-58)
RM2	Mollusk	5 (0-20)	10 (0-33)	**73 (50-100)**	12 (0-39)
	Crustacean	26 (0-48)	**37 (0-77)**	5 (0-21)	32 (0-98)
	Fish	5 (0-16)	33 (0-84)	7 (0-22)	**55 (0-100)**
RM3	Mollusk	18 (0-41)	11 (0-47)	**54 (0-87)**	17 (0-100)
	Crustacean	3 (0-21)	**79 (1-100)**	6 (0-25)	12 (0-84)
	Fish	3 (0-15)	**71 (24-100)**	3 (0-15)	23 (0-62)
RefM	Crustacean	**57 (36-83)**	13 (0-41)	20 (1-42)	10 (0-35)
	Fish	**53 (34-73)**	18 (0-51)	15 (0-31)	14 (0-43)
Dry season	Consumer	C_3_ plants	Phytoplankton	Seagrass	Epiphytes
RM1	Crustacean	32 (7-56)	**38 (0-82)**	6 (0-21)	24 (0-67)
	Fish	26 (12-41)	**34 (0-74)**	8 (0-24)	32 (0-76)
RM2	Crustacean	20 (0-38)	**49 (0-100)**	4 (0-18)	27 (0-79)
	Fish	1 (0-9)	**88 (52-100)**	2 (0-9)	9 (0-42)
RM3	Mollusk	**42 (27-55)**	9 (0-32)	**41 (23-55)**	8 (0-35)
	Crustacean	4 (0-22)	33 (0-100)	4 (0-20)	**59 (0-100)**
	Fish	4 (0-17)	**70 (23-95)**	6 (0-17)	21 (0-63)
RefM	Crustacean	32 (0-72)	**42 (0-100)**	11 (0-32)	15 (0-50)
	Fish	22 (0-54)	**51 (2-100)**	11 (0-30)	16 (0-52)

**Notes.**

RM1 mangrove restored in 2010 RM2 mangrove restored in 2014 RM3 mangrove restored in 2018 RefM reference mangrove

In the “nortes” season, the contribution of seagrass decreased and that of phytoplankton increased (Table 3). The consumers of RM1 assimilated phytoplankton as main basal resource (57% and 59%), and epiphytes were a secondary carbon resource (18% and 17%). In RM2, the main resources assimilated were seagrass for mollusks (73%); phytoplankton for crustaceans (37%), and epiphytes for fish (55%). The second most assimilated carbon resource was epiphytes for mollusk and crustaceans (12% and 32%, respectively) and phytoplankton for fish (33%). In RM3, the mollusks mainly assimilated seagrass (54%); while crustaceans and fish assimilated mainly phytoplankton (79% and 71%, respectively). The second most assimilated carbon resource were C_3_ plants for mollusks (18%), and epiphytes for crustaceans and fish (12% and 23%, respectively). In the reference mangrove, C_3_ plants were the main resource sustaining the communities (57% and 53%), the secondary carbon resource was seagrass for crustaceans (20%) and phytoplankton for fish (18%).

In the dry season, almost all consumers in all areas (restored and reference) assimilated mainly phytoplankton, on average from 34% to 88% (Table 3). In RM1, C_3_ plants were a secondary carbon source for crustaceans (32%) and epiphytes for fish (32%). In RM2, epiphytes were the secondary carbon resource for the consumers (27% and 9%). In RM3, mollusk assimilated mainly C_3_ plants (42%), follow by seagrass (41%); while crustaceans assimilated mainly epiphytes (59%) follow by phytoplankton (33%). In the reference mangrove, C_3_ plants were the secondary source assimilated by consumers (32% and 22%).

### Trophic niche

**Figure 5:**
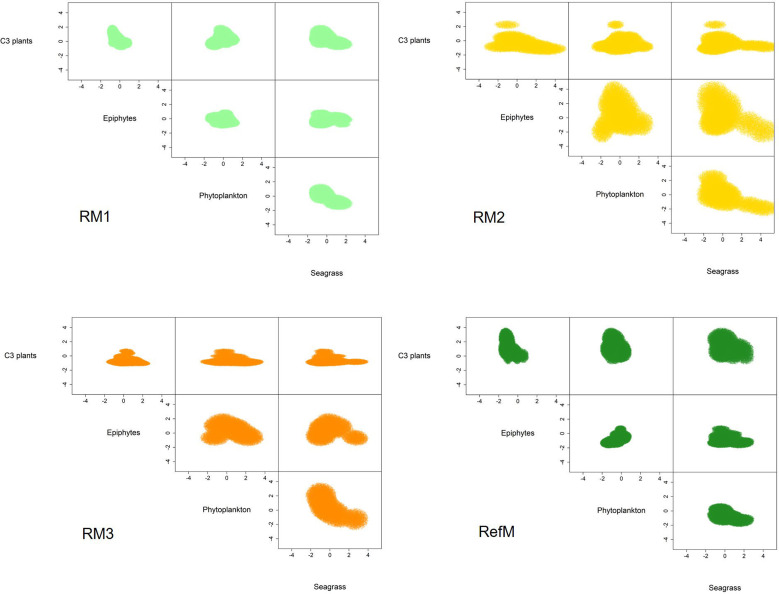
Trophic niche hypervolumes of consumers in restored and reference mangroves. Axes represent z-cores of estimated dietary contributions. RM1, mangrove restored in 2010; RM2, mangrove restored in 2014; RM3, mangrove restored in 2018; RefM, reference mangrove.

The mangrove area with the longest time of restoration (RM1) had a trophic niche hypervolume of 21.3 similar to the reference mangrove of 36.1 (Fig. 5). They had a 49% overlap (Sorensen similarity = 0.49, CI = 0.22–0.5) with similar plants C_3_ consumption (Supplementary material S2, Fig. 2). The trophic niche of RM2 was larger (173.1), based on the consumption of epiphytes, phytoplankton, and seagrass. This area had a 19% overlap with the reference mangrove (Sorensen similarity = 0.19, CI = 0.03–0.27). The trophic niche of the area with the most recent restoration (RM3) was 50.2, with consumption similar of RM2, and had a 17% overlap with the reference mangrove (Sorensen similarity = 0.17, CI = 0.01–0.21).

## Discussion

This study found that food web structure in the restored and reference mangroves areas varied seasonally, depending on restoration time and area location. The Layman et al. (2007) metrics and SEA_C_ confirmed that the reference mangrove (RefM) had higher trophic diversity than the restored areas (Table 2, Fig. 4). As hypothesized, the area with the longest restoration time (RM1) had a higher trophic diversity value and Layman et al. (2007) metrics than the other restored areas (Table 2). The mixing models estimated seasonal variability in assimilated resources (Table 3). Seagrass and epiphytes were the most important resources for maintaining food webs in the rainy season. Phytoplankton and epiphytes were the main resources in restored mangroves in the “nortes” season, while C_3_ plants (mangroves) were the most assimilated basal resource in the reference mangrove area (RefM). Phytoplankton was the most important resource in the dry season for almost all consumers. These changes are the result of seasonal patterns of primary producers in the Términos Lagoon (Yáñez Arancibia et al., 2013; Herrera-Silveira et al., 2019) and indicates the importance of connectivity for the input of allochthonous resources from adjacent marine environments into the mangrove ecosystems (Connolly, Gorman & Guest, 2005; Bouillon et al., 2007; Igulu et al., 2013). The trophic niche (hypervolume) indicated that the energy flow in the restored mangroves areas had two different responses. The oldest restored mangrove niche (RM1) was the most similar to the reference mangrove and had the greatest overlap (Fig. 5, Supplementary material S2, Fig. 2), indicating a better level of recovery of the food web (James et al., 2020).

As expected, the studied mangrove areas exhibited environmental changes according to regional seasonality (Yáñez Arancibia et al., 2013; Herrera-Silveira et al., 2019). Notable among these changes were greater water depth and lower DO in the rainy season (Fig. 2, Table 1). Maximum flooding occurs during rainfall (Yáñez Arancibia et al., 2013), which carries organic matter that decomposes and causes oxygen depletion in the water (Ruiz-Marín et al., 2009). Flooding during this season and incorporation of nutrients into the ecosystem increased macroinvertebrate (mollusks and crustaceans) abundance in mangrove ecosystems (Lee, 2008; Salimi et al., 2021). Lower water temperatures occurred during the “nortes” season (Fig. 2, Table 1), caused by cold rains from polar air masses (Yáñez Arancibia et al., 2013; Herrera-Silveira et al., 2019). The organic matter and nutrients brought to the ecosystem by the rains promoted phytoplankton production in the estuaries (Day Jr et al., 1988), which was maintained as temperatures increased (Rivera-Monroy et al., 1998). As is generally the case in mangrove ecosystems, TDS concentration, conductivity and salinity increased notably during the dry season (Fig. 2, Table 1). Salinity is particularly important as it is a determining factor for the entry of marine fish into the mangrove habitats, increasing their abundance and distribution (Ley, McIvor & Montague, 1999; Faunce & Serafy, 2006).

Trophic niche (SEA_C_) values varied between areas and seasons in response to resource availability and environmental conditions. However, trophic diversity was higher in the reference mangrove (RefM) compared to the restored areas (Fig. 4). The area with the longest restoration time (RM1), exhibited the broadest trophic niche (TA) and more carbon sources (dC) than the other restored areas (RM2 and RM3) (Table 2). Restored ten years prior to sampling, RM1 had the largest mangrove trees and amount of foliage among the three restored areas, which is a characteristic sign of successful restoration (Pérez-Ceballos et al., 2017). The reestablishment of hydrological connectivity allows the transfer of matter and energy to the mangroves (Valentine-Rose & Layman, 2011), as well as the recolonization of macrofauna such as mollusks and crustaceans and the successive increase of aquatic fauna (Bosire et al., 2008; Salmo III, Tibbetts & Duke, 2018).

The mixing models indicated that food webs varied seasonally and relied mainly on carbon sources of allochthonous origin (Table 3). Both seagrasses and epiphytes contributed most to the organic C pool in the rainy season. The importance of seagrass as a main carbon source for mollusks from all restoration mangrove areas and all consumers of reference mangrove (Table 3) was highlighted. Seagrass did not grow in the mangrove areas studied, but the inland side of Carmen Island harbors vast areas of seagrass composed of three species: *Thalassia testudinium* as dominant, with *Halodule wrigtii* and *Syringodium filiforme* (Moore & Wetzel, 1988). Their maximum productivity occurs in the dry season, promoted by high transparency and salinity (Moore & Wetzel, 1988; Yáñez Arancibia et al., 2013), and is incorporated into the food webs in rainy season with a delay. The entry of seagrass into the channels is due to inwelling that enters the system as particulate matter or detritus (Connolly, Gorman & Guest, 2005; Santos et al., 2021), transported by ocean currents and tides (Bouillon et al., 2007). It is also favored by the movement of the organisms between connected coastal ecosystems. In these systems, many fish species’ use of mangroves is linked to seagrass beds in Términos Lagoon (Yáñez Arancibia, Lara-Domínguez & Day Jr, 1993).

Epiphytes were an important source of carbon for crustaceans and fish in all restored areas during rainy season, as well as the secondary carbon source in the “nortes” season (Table 3). Seagrasses serve as substrate for the development of epiphytes, and the production of epiphytes has been shown to reach about 20% of the production of seagrasses (Van Montfrans, Wetzel & Orth, 1984). The food webs associated with seagrasses depend on epiphytes as a main source of carbon, contributing *via* detrital patyways and showing seasonal changes in their assimilation (James et al., 2022). Thus, seagrasses constitute an important direct and indirect (epiphytes pathway) energy source for the mangrove communities analyzed.

Phytoplankton was the main basal resource assimilated by macroinvertebrates of restored mangrove areas in the “nortes” season and by almost all consumers in the dry season (Table 3). Regardless of their status in the mangrove (*i.e.,* resident, occasional visitor, or seasonal visitor), all fish depended on the development of phytoplankton in mangrove areas (Supplementary Material S1, T4). Phytoplankton is an important source of carbon in mangrove ecosystems (Kristensen et al., 2008); in mangrove restoration areas it is an important basal source assimilated by macroinvertebrate epifauna and infauna (Van Hieu et al., 2020; Then et al., 2021). In Términos Lagoon it is a highly available resource. High phytoplankton biomass has been recorded in the mangrove zones (estuaries) during low water levels (Day Jr et al., 1988), and high productivity in the lagoon is recorded in the rainy and “nortes” seasons (Day Jr et al., 1988; Herrera-Silveira et al., 2019). Likewise, the lagoon sediment has a high concentration of phytoplankton (Gonneea, Paytan & Herrera-Silveira, 2004). The assimilation of it by consumers occurs with a delay at maximum productivity (Day Jr et al., 1988), highlighting the importance of connectivity between Términos Lagoon and the Gulf of Mexico and the increased seawater incursion into the system facilitated by mangrove restoration.

As expected, the consumers in the reference mangrove (RefM) assimilated a greater proportion of C_3_ plants (mangrove) than in the restored areas; the contribution of mangroves was a primary (dry season) and secondary source of carbon (rainy and “nortes” seasons). In contrast, mangroves made smallest contribution as a basal carbon resource in consumers in the restored areas, except by mollusks in the area with the most recent time since restoration (RM3), and crustaceans of RM1 in dry and rainy seasons (Table 3). The consumption and assimilation of mangrove leaves is an important resource for some mangrove ecosystems (Abrantes et al., 2015; Abrantes & Sheaves, 2008; Mendoza-Carranza et al., 2010; Muro-Torres et al., 2020). Its assimilation is considered limited for some species such as invertebrates, due to its low nutritional level and poor digestibility (Cannicci et al., 2008; Lee et al., 2014). It has also been considered that their contribution to food webs is greater in closed environments, where there is little input from allochthonous carbon sources (Bouillon, Connolly & Lee, 2008). Our results indicate that the role of reference mangrove is not only to provide refuge for fish from predators (Nagelkerken et al., 2008). Mangroves in this area also supply food for both resident and mobile species like transient fish.

The trophic niche shows that food webs in the restored mangroves displayed two responses in terms of basal resource use. The more recently restored mangroves areas (RM2 and RM3) had larger niches; and increased their trophic niche and carbon source based mainly on the assimilation of seagrasses, epiphytes, and phytoplankton, especially the consumers in RM2 (Fig. 5). Both also had little niche overlap with the reference mangrove (RefM) (Supplementary material S2, Fig. 2). The mangrove area with the longest restoration period (RM1) had a similar niche to the reference one (Fig. 5) and a greater overlap. Since the same restoration techniques were used (Pérez-Ceballos et al., 2020), the variation found may be due to the location, configuration of the area and restoration time. The mangrove area restored in 2014 (RM2) is located at a shorter distance from Términos Lagoon and the mangrove area restored in 2018 (RM3) is in contact with a wider channel (Fig. 1). In both cases, this may facilitate greater entrance of marine water and input of allochthonous basal sources (Connolly, Gorman & Guest, 2005; Bouillon et al., 2007; Igulu et al., 2013). Due to its proximity to Términos lagoon, the restored mangrove area (RM2) had more influx of seasonal or occasional transient marine fish that feed in the lagoon (Amador del Ángel, Guevara Carrió E del & Lastra Santiago, 2007), such as juvenile snook (*Centropomus undecimalis*) and snapper (*Lutjanus griseus*) (Guevara et al., 2007; Aragón-Flores et al., 2022).

The response and recovery of restored mangrove areas will depend on the success of mangrove tree restoration (Pérez-Ceballos et al., 2017), the location and configuration of the area (Van Hieu et al., 2020; Then et al., 2021), and consumer colonization over time (Bosire et al., 2008; Salmo III, Tibbetts & Duke, 2018), among other factors. In our analysis, only the oldest restored mangrove area (RM1) had a similar trophic niche response compared to the reference area (Fig. 5) and showed major overlap (49%). As a result of the restoration process, after almost 10 years, the structure and function observed in this mangrove area is like that observed in the reference mangrove, which is one of the objectives of the restoration (Bosire et al., 2008; Moreno-Mateos et al., 2020). Maybe this response was obtained due to the location of the system, which is surrounded by natural mangroves (Fig. 1) as well as the time elapsed since restoration, which have allowed the recolonization of macroinvertebrates and fish. For example, the presence and abundance of sesarmid crabs (*Aratus pisonii*) is considered a restoration success. Because these crabs are herbivores and consume mangrove leaves, they are involved in the processing of organic matter and are considered important in the structure and functioning of the mangrove ecosystems. Crabs of the genus *Uca* have also shown a strong dependence on mangroves (Cannicci et al., 2008). The similarity between trophic niches value is high compared to that reported in other restored coastal ecosystems (James et al., 2020), and it is an indicator that food web function is being recovered and the success of restoration so far.

Our results provide an approximation of the interactions and dynamics of the communities in restoration areas; allowing us to determine which are the main resources that maintain the consumers, as well as to understand that the flow of nutrients and the trophic dynamics vary seasonally depending on the time of restoration and geographic location. We detected that food webs varied seasonally like other coastal ecosystems (Abrantes et al., 2015; James et al., 2022). As a result of the restoration process, connectivity with the Términos Lagoon was restored, allowing the entry and maintenance of the resources to the aquatic communities. This study supports the idea that mangrove ecosystems receive important carbon sources from nearby environments to maintain the food webs (Connolly, Gorman & Guest, 2005; Bouillon, Connolly & Lee, 2008; Kruitwagen et al., 2010; Igulu et al., 2013). In addition, the C_3_ plants (mangroves) were an important source of carbon for the reference mangrove area throughout the seasons (Abrantes et al., 2015; Abrantes & Sheaves, 2008; Mendoza-Carranza et al., 2010; Muro-Torres et al., 2020). As a result of the restoration process, the structure and function of food webs was re-established but it produced two responses: an increase of trophic and carbon sources diversity (RM2 and RM3), and the use of carbon resource (RM1) as a reference mangrove. The present results therefore suggest that ecosystem composition, diversity, and ecological functions can be restored over time like other restored mangroves ecosystems.

## Conclusions

Due to the accelerated loss of mangrove ecosystems, several restoration projects have been developed to recover their structure and function. Using stable isotopes *δ*^13^C and *δ*^15^N, we identified that the area with more restoration time (10 years) had greater trophic diversity and a trophic niche similar to the reference mangrove area. Environmental characteristics and resource availability for consumers varied seasonally. The availability and diversity of the assimilated resources varied seasonally and is linked to primary productivity in Términos Lagoon. Although the functional response to the restoration process varies in the areas analyzed, our results highlight the importance of connectivity with the Lagoon Términos for the input of resources that maintain food webs. Further evaluation of estimating the abundance, richness, and density of macroinvertebrates (mollusks and crabs) as indicators of restoration success is recommended, since they were the taxonomic groups that showed the greatest differentiation in the assimilation of resources between mangrove restoration stages (Van Hieu et al., 2020; Then et al., 2021). We also suggest that future work should estimate the assimilation of microphytobenthos, a carbon source that develops in mangroves (Bouillon, Connolly & Lee, 2008; Nagelkerken et al., 2008). Ongoing evaluation of trophic diversity and resources in mangroves *via δ*^13^C and *δ*^15^N values as change descriptors is recommended over the medium-term to monitor ecosystem health and recovery, especially with estimators like trophic niche (James et al., 2020).

##  Supplemental Information

10.7717/peerj.15422/supp-1 Supplemental Information 1 Supplementary Tables

10.7717/peerj.15422/supp-2 Supplemental Information 2 Supplementary Figures

## Additional Information and Declarations

### Data Availability

The following information was supplied regarding data availability: The raw measurements are available in the Supplemental Tables.
