# Supplementary material for: Assessment of aquatic food web and trophic niche as a measurement of recovery function in restored mangroves in the Southern Gulf of Mexico
Source: PeerJ. 2023 Jun 6;11:e15422. doi: 10.7717/peerj.15422 (PMC10252823; doi:10.7717/peerj.15422)

**Supplementary material 2**

**Assessment of aquatic food web and trophic niche as a measurement of recovery function in restored mangroves in the Southern Gulf of Mexico**

Miriam Soria-Barreto^1,2^, Rosela Pérez-Ceballos^3^, Arturo Zaldívar-Jiménez^4^, Rolando Gelabert Fernández^1^

^1^ Centro de Investigación de Ciencias Ambientales, Facultad de Ciencias Naturales, Universidad Autónoma del Carmen, Ciudad del Carmen, Campeche, Mexico

^2^ Present address: Laboratorio de Ecología Acuática y Monitoreo Ambiental, CEDESU, Universidad Autónoma de Campeche, San Francisco de Campeche, Campeche, Mexico

^3^ CONACYT Instituto de Ciencias del Mar y Limnología Estación El Carmen, Universidad Nacional Autónoma de México, Ciudad del Carmen, Campeche, Mexico

^4^ ATEC Asesoría Técnica y Estudios Costeros SCP, Mérida, Yucatán, Mexico

Corresponding Author:

Miriam Soria-Barreto

Av. Héroe de Nacozari No. 480, 24070, San Francisco de Campeche, Campeche, Mexico

Email address: mmsoriab@gmail.com

**Fig. S1**. Simulated mixing region for stable isotope signatures of consumers (open circles) and sources (black squares) during seasons. The probability contour is 95% level, consumers located outside of this region were not considered in the mixing models. s1 = C_3_ plants, s2 = phytoplankton, s3 = seagrass, s4= epiphytes, RM1 = mangrove restored in 2010, RM2 = mangrove restored in 2014, RM3 = mangrove restored in 2018; RefM = reference mangrove.

1. Rainy season.

RM1

RM2

RM3

RefM

1. “Nortes” season.

RM1

RM2

RM3

RefM

1. Dry season.

RM1

RM2

RM3

RefM

**Fig. S2**. Trophic niche hypervolumes of consumers in reference mangrove vs. restored mangrove areas. Axes represent z-scores of estimated basal sources contributions.

1. Restored mangrove 1 vs. Reference mangrove.


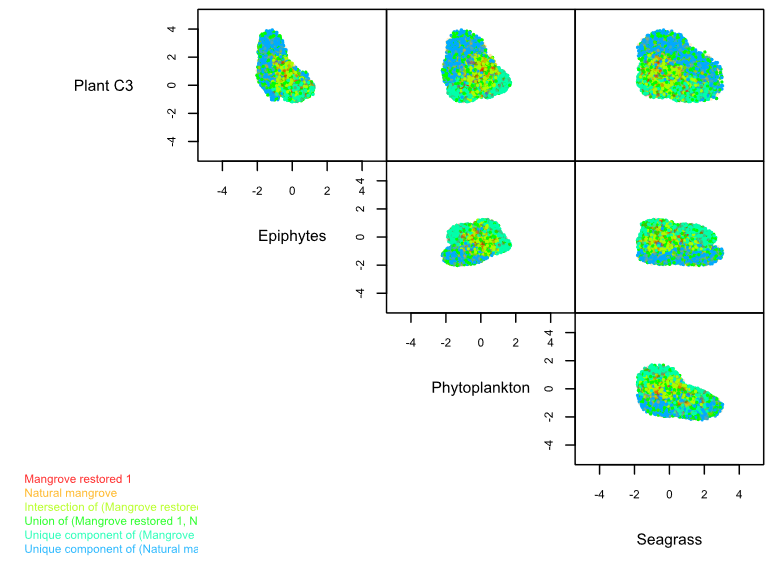


1. Restored mangrove 2 vs. Reference mangrove.


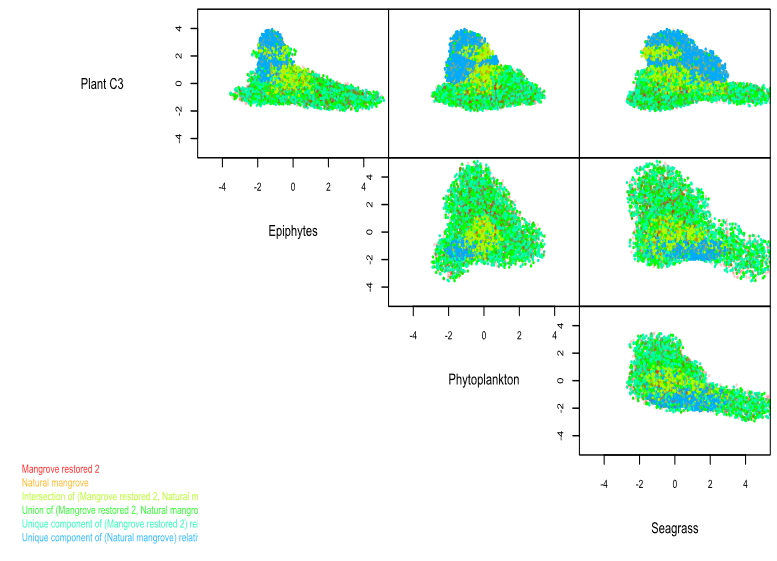


1. Restored mangrove 3 vs. Reference mangrove.


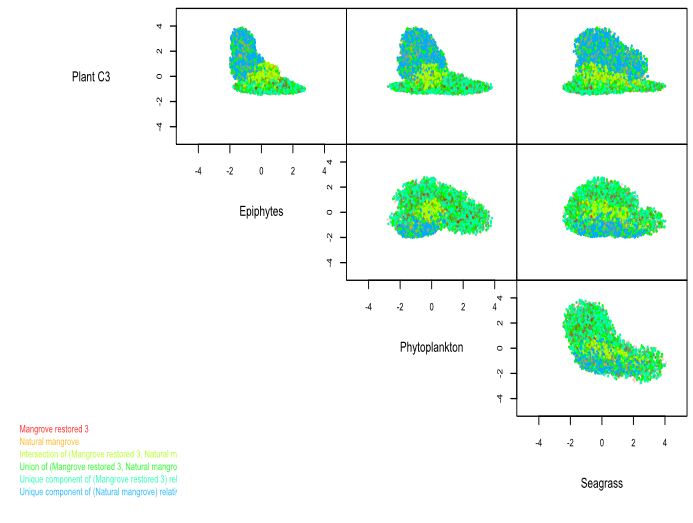

Supplement: Supplemental Information 2 [file peerj-11-15422-s002.docx]
